# Supplementary material for: In-silico platform for the multifunctional design of 3D printed conductive components
Source: Nat Commun. 2025 Feb 4;16:1359. doi: 10.1038/s41467-025-56707-y (PMC11794641; doi:10.1038/s41467-025-56707-y)
Supplement: Supplementary file 1 — Supplementary Information [file 41467_2025_56707_MOESM1_ESM.pdf]

Supplementary Information: *In-silico* tool for the multifunctional design of 3D printed conductive components

Javier Crespo-Miguel[1], Sergio Lucarini[2,3,4], Sara Garzon-Hernandez[1], Angel Arias[1], Emilio Martinez-Pañeda[4,5], and Daniel Garcia-Gonzalez[1,*]

[1]Department of Continuum Mechanics and Structural Analysis, University Carlos III of Madrid, Avda. de la Universidad 30, 28911 Leganés, Madrid, Spain
[2]BCMaterials, Basque Center for Materials, Applications and Nanostructures, UPV/EHU Science Park, 48940 Leioa, Spain
[3]Ikerbasque, Basque Foundation for Science, 48009 Bilbao, Spain
[4]Department of Civil and Environmental Engineering, Imperial College of London, South Kensington Campus London, SW7 2AZ
[5]Department of Engineering Science, University of Oxford, Oxford OX1 3PJ, UK
[*]Corresponding author: danigarc@ing.uc3m.es

Mesoscopic full-field homogenised formulation

In this section, we present the full-field homogenisation formulation of the mechano-electrical response of 3D printed PLA/CB composites at the mesoscopic scale. This model aims to obtain the homogenised material properties of the printed samples depending on mesostructural features (e.g., inter-layer/inter-filament adhesion planes or voids direction and size). To this end, the governing equations of the problem are solved in a Representative Volume Element (RVE) of the mesostructure, considered as the union of four filaments.

Mechanical behaviour of constituent phases

The mechanical constitutive behaviour of the pure filament and adhesion zones was modelled with an elasto-viscoplastic rheological model. We decompose the deformation gradient into an elastic component, $\mathbf{F}^{\mathrm{e}}$, and a plastic one, $\mathbf{F}^{\mathrm{p}}$, as:

$$\mathbf{F} = \mathbf{F}^{\mathrm{e}} \cdot \mathbf{F}^{\mathrm{p}} \ . \tag{1}$$

Due to the high brittleness of conductive PLA/CB composite compared to pure PLA, the hardening caused by the network resistance of the polymer at high strains can be neglected [1]. The proposed

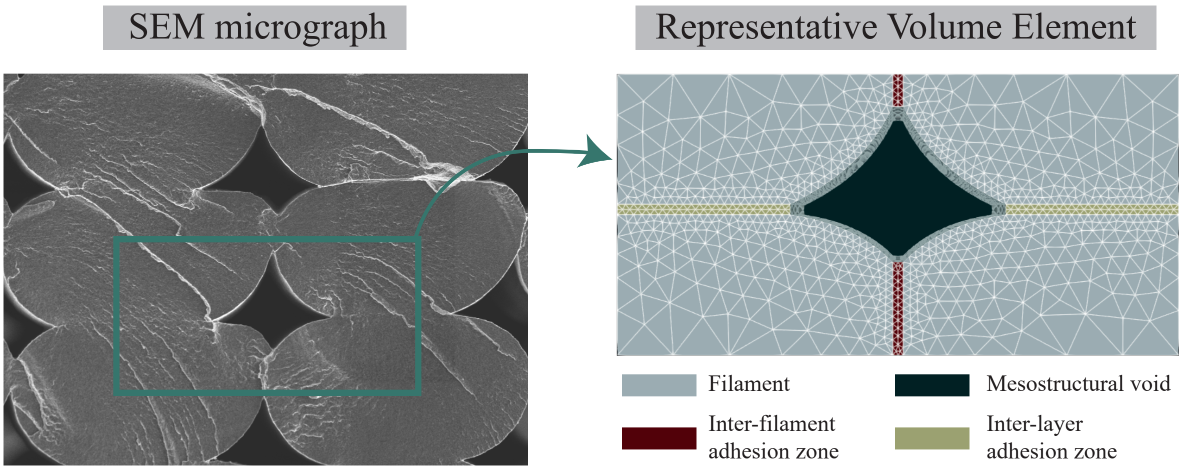

Supplementary Figure 1. Diagram of the four phases of the mesoscopic RVEs.

rheological model consists of a unique branch, with a hyperelastic spring in series with a viscoplastic dashpot. The considered energy strain function is based on a compressible Neo-Hookean model, obtaining the following Piola-Kirchhoff stress:

$$\mathbf{P}(\mathbf{F},\mathbf{F}^{\mathrm{p}}) = [\mu(\mathbf{B}^{\mathrm{e}}-\mathbf{I}) + \lambda ln(J_F^{\mathrm{e}})\mathbf{I}]\cdot\mathbf{F}^{-T} \ , \tag{2}$$

where μ and λ are the Lamé constants, $\mathbf{B}^{\mathrm{e}} = \mathbf{F}^{\mathrm{e}}(\mathbf{F}^{\mathrm{e}})^T$, $J_F^{\mathrm{e}} = det(F^{\mathrm{e}})$ and $\mathbf{I}$ is the second order identity tensor.The evolution of the plastic deformation $\mathbf{F}^{\mathrm{p}}$ is governed by a yield function with plastic hardening defined as:

$$f = \overline{\sigma} - \sigma_{\mathrm{Y}} - k \ , \tag{3}$$

where σ_{Y} is the yield stress and $\overline{\sigma} = \sqrt{3J_2}$ with:

$$J_2 = \frac{1}{2}\boldsymbol{\sigma}^{\mathrm{dev}} : \boldsymbol{\sigma}^{\mathrm{dev}} \ . \tag{4}$$

Where σ_{dev} denotes the deviatoric part of the Cauchy stress tensor, defined as the difference between the total stress and its hydrostatic part ($\boldsymbol{\sigma}_{\mathrm{dev}} = \boldsymbol{\sigma} - (tr(\boldsymbol{\sigma})/3)\mathbf{I}$). The hardening law k is defined as:

$$k = (\sigma_{\mathrm{s}} - \sigma_{\mathrm{Y}})[1 - exp(-H\xi^{\mathrm{p}})] \ , \tag{5}$$

where $\xi^{\mathrm{p}} = \sqrt{\frac{2}{3}\mathbf{e}^{\mathrm{p}} : \mathbf{e}^{\mathrm{p}}}$ is the equivalent plastic strain, that depends on the plastic deviatoric deformation tensor $\mathbf{e}^{\mathrm{p}} = \mathrm{dev}\left(1/2\left(\mathbf{F}^{\mathrm{p}}\cdot(\mathbf{F}^{\mathrm{p}})^T - \mathbf{I}\right)\right)$. σ_{s} is the saturation stress and H is the hardening parameter. The plastic surface derives from:

$$\mathbf{N} = \frac{\partial f}{\partial \boldsymbol{\sigma}} = \frac{\boldsymbol{\sigma}^{\mathrm{dev}}}{\sqrt{\frac{4}{3}J_2}} \ . \tag{6}$$

The plastic strain rate is defined as:

$$\dot{\bar{\varepsilon}}^{\mathrm{p}} = \begin{cases} \dot{\varepsilon}_{\mathrm{o}}(exp[\frac{1}{C}(\frac{\overline{\sigma}}{\sigma_{\mathrm{Y}}+k}) - 1] - 1) & f > 0 \\ 0 & f <= 0 \end{cases} \tag{7}$$

with C being a model parameter describing the strain rate dependency and $\dot{\varepsilon_o}$ the reference strain rate. The former parameter is calibrated using experimental data obtained from uniaxial tensile tests performed to PLA/CB filament samples at three different strain rates (10^{-4},10^{-3} and 10^{-2} s^{-1}). The latter parameter is defined as the lowest strain rate used in those experiments (10^{-4} s^{-1}). The time variation of the plastic deformation gradient follows:

$$\dot{\mathbf{F}^{\mathrm{p}}} = (\mathbf{F}^{\mathrm{e}})^{-1}\cdot\dot{\bar{\varepsilon}}^{\mathrm{p}}\mathbf{N}\cdot\mathbf{F} \ . \tag{8}$$

The experimental results show weaker mechanical properties of the adhesion interface with respect to the filament itself. To model this, the mechanical parameters of adhesion zones are initially degraded taking as reference the ones obtained for the pure filament. This degradation was set by the parameter γ for the inter-filament adhesion and η for the inter-layer adhesion, both smaller than 1. The degradation applies to yield stress and Young's modulus, following:

$$\sigma_{\mathrm{Y}}^{\text{ref,inter-fil}} = (1-\gamma)\sigma_{\mathrm{Y}}^{\text{ref,fil}} \ ; \ \sigma_{\mathrm{Y}}^{\text{ref,inter-lay}} = (1-\eta)\sigma_{\mathrm{Y}}^{\text{ref,fil}}, \tag{9}$$

$$E^{\text{ref,inter-fil}} = (1-\gamma)E^{\text{ref,fil}} \ ; \ E^{\text{ref,inter-lay}} = (1-\eta)E^{\text{ref,fil}}, \tag{10}$$

where $\sigma_{\mathrm{Y}}^{\text{ref,inter-fil}}$, $\sigma_{\mathrm{Y}}^{\text{ref,inter-lay}}$ and $\sigma_{\mathrm{Y}}^{\text{ref,fil}}$ are the yield stress at reference temperature of the inter-filament adhesion zone, inter-layer adhesion zone and filament, respectively. $E^{\text{ref,inter-fil}}$, $E^{\text{ref,inter-lay}}$ and $E^{\text{ref,fil}}$ are the Young's modulus at reference temperature of the inter-filament adhesion zone, inter-layer adhesion zone and filament, respectively (see Supplementary Fig. 1 for a schematic representation of each phase). The mesostructural voids were modelled and meshed but, due to their null stiffness, a negligible Young's modulus was defined in this phase. The elasto-viscoplastic parameters of the three phases are detailed in Supplementary Table 1 whereas the thermo-mechanical parameters are detailed in Supplementary Table 5.

Electrical behaviour of constituent phases

From an electrical perspective, every phase was considered to have an electrical conductivity Σ defining the relation between the electric current density and electric field as:

$$\mathbb{j} = \Sigma \mathbb{e}, \tag{11}$$

where $\mathbb{j}$ is the electric current density and $\mathbb{e}$ is the electric field, both of them in the current configuration. In conductive polymer composites, the distance between the particles is crucial, as it enables the creation of conductive paths in the composite. To model this dependence, the relation of conductivity with mechanical deformation was set as linearly dependent with the first invariant of deformation gradient, I_1^F (see [2]):

$$\Sigma(\mathbf{F}) = \Sigma_{ref}(1 - C_F(I_1^F - 3)), \tag{12}$$

where C_F is the strain sensitivity parameter of the conductivity.
Analogously to the degradation of the mechanical properties in adhesion zones, the same is set for the electrical parameters. In this case, we define a logarithmic degradation[1] of properties using the parameter α for the inter-filament adhesion and β for the inter-layer adhesion, both smaller than 1.

$$\Sigma^{\text{ref,inter-fil}} = exp(\alpha ln(\Sigma^{air}) + ln(\Sigma^{\text{ref,fil}}))\ ;\ \Sigma^{\text{ref,inter-lay}} = exp(\beta ln(\Sigma^{air}) + ln(\Sigma^{\text{ref,fil}}))\ , \tag{13}$$

where Σ^{air}, $\Sigma^{\text{ref,inter-fil}}$ and $\Sigma^{\text{ref,inter-lay}}$ are the conductivity of the air (i.e., mesostructural void), inter-filament adhesion zone, inter-layer adhesion zone and filament, respectively.

Homogenisation framework and governing equations

The postulated full-field homogenisation finite element framework accounts for finite deformations. The different outcomes (i.e., electrical and mechanical) depend on the deformed shape and volumetric changes, defined in terms of the deformation gradient:

$$\mathbf{F}(\mathbf{x}) = \mathbf{I} + \nabla_{\mathbf{X}}\mathbf{u}\ . \tag{14}$$

Considering periodic fields and boundary conditions, the total displacement field $\mathbf{u}(\mathbf{X})$ can be decomposed into a macroscopic variation and a fluctuating displacement field $\widetilde{\mathbf{u}}(\mathbf{X})$ as:

$$\mathbf{u} = (\overline{\mathbf{F}} - \mathbf{I}) \cdot \mathbf{X} + \widetilde{\mathbf{u}}\ , \tag{15}$$

where $\overline{\mathbf{F}}$ is the macroscopic deformation gradient and $\mathbf{X}$ is the position in the reference configuration. Substituting the previous equation into Eq. 14 and discarding second-order terms:

$$\mathbf{F} = \nabla_{\mathbf{X}}\mathbf{u} + \mathbf{I} = \nabla_{\mathbf{X}}[(\overline{\mathbf{F}} - \mathbf{I}) \cdot \mathbf{X} + \widetilde{\mathbf{u}}] + \mathbf{I} = \overline{\mathbf{F}} + \nabla_{\mathbf{X}}\widetilde{\mathbf{u}}\ . \tag{16}$$

To solve the mechanical problem, the linear momentum balance is described in the reference configuration as:

$$\nabla_{\mathbf{X}} \cdot \mathbf{P} = \mathbf{0} \quad \forall \mathbf{X} \in \Omega_0\ , \tag{17}$$

where the first Piola-Kirchhoff stress field $\mathbf{P}$ is defined by the different constituent phases inside the RVE. Typically, a strain-controlled test is imposed via the macroscopic deformation gradient $\overline{\mathbf{F}}$. To impose the stress-controlled components macroscopically, the spatial average Piola-Kirchhoff stress tensor components are enforced to be equal to the imposed ones. This imposition is set using a Lagrange multiplier for each stress component to be controlled:

$$\left(\frac{1}{|\Omega_0|} \int_{\Omega_0} \mathbf{P}\left(\mathbf{F}, \mathbf{F}^p, \theta, \mathbf{X}\right) \mathrm{d}\Omega_0 - \overline{\mathbf{P}} \right) : \boldsymbol{\lambda}_m = 0\ . \tag{18}$$

This mechanical Lagrange multiplier matrix modifies the original expression of the solution deformation gradient as:

$$\mathbf{F} = \overline{\mathbf{F}} + \nabla_{\mathbf{X}}\widetilde{\mathbf{u}} + \boldsymbol{\lambda}_m\ . \tag{19}$$

Analogously with the mechanical contribution, the electric potential field ϕ can be decomposed into a macroscopic contribution and a fluctuating electric potential field $\widetilde{\phi}$ as:

$$\phi = \nabla_{\mathbf{X}}\overline{\phi} \cdot \mathbf{X} + \widetilde{\phi} = -\overline{\mathbb{E}} \cdot \mathbf{X} + \widetilde{\phi}\ , \tag{20}$$

[1] The changes in orders of magnitude for electrical conductivity are more likely to occur than for Young's modulus or yield stress. Thus, using a linear degradation would have led to values of α and β close to 1.

where $\overline{\mathbb{E}}$ is the (imposed) macroscopic electric field. The microscopic (solution) electric field reads as:

$$\mathbb{E} = -\nabla_{\mathbf{X}}\phi = \nabla_{\mathbf{X}}\overline{\mathbb{E}} \cdot \mathbf{X} + \overline{\mathbb{E}} \cdot \nabla_{\mathbf{X}}\mathbf{X} - \nabla_{\mathbf{X}}\widetilde{\phi} = \overline{\mathbb{E}} - \nabla_{\mathbf{X}}\widetilde{\phi} \ . \tag{21}$$

The governing equations for the electric problem correspond to the Maxwell equations (in the absence of magnetic field):

$$\nabla_{\mathbf{X}} \cdot \mathbb{D} = Q \quad ; \quad \nabla_{\mathbf{X}} \times \mathbb{E} = 0 \quad ; \quad \nabla_{\mathbf{X}} \cdot \mathbb{J} = 0 \tag{22}$$

with Q being the charge accumulated within the body and $\mathbb{D}$ the electric displacement. Note that the second equation in 22 is imposed by defining the electric field as $\mathbb{E} = -\nabla_{\mathbf{X}}\phi$, and the third equation is obtained by applying the divergence operator to Ampere's circuital law and assuming $\nabla_{\mathbf{X}} \cdot \epsilon_0 \frac{\partial \mathbb{E}}{\partial t} = \frac{\partial Q}{\partial t} = 0$. By performing a pull-back of the Eulerian electric current density ($\mathbb{j}$) and electric field ($\mathbb{e}$), the relation between those variables at the spatial and material configurations can be written as:

$$\mathbb{J} = J_F \mathbf{F}^{-1} \cdot \mathbb{j} \quad ; \quad \mathbb{E} = \mathbf{F}^T \cdot \mathbb{e} \ . \tag{23}$$

Thus, the electric current density and electric field in the reference configuration can be related as follows:

$$\mathbb{J} = \Sigma J_F \mathbf{F}^{-1} \cdot \mathbf{F}^{-T} \cdot \mathbb{E} \ . \tag{24}$$

By considering this relation between the electric field and electric current density, as well as the split shown in Eq. 20 and Eq. 21, it is possible to solve the electrical governing equation in the reference configuration, Eq. 22.

Gathering the previous strong form formulations, we can summarise the residual expressions used to solve the coupled non-linear system of equations in our finite element model, which consists of the periodic RVE and periodic boundary conditions. From a mechanical perspective, we account for the mechanical equilibrium, Eq. 25, the residual of the plastic deformation gradient (as we consider it as an independent variable), Eq. 26, and the residual of the mechanical Lagrange multiplier, Eq. 27:

$$\mathbf{R}^m = \int_{\Omega_0} \mathbf{P}(\mathbf{F}, \mathbf{F}^p, \theta, \mathbf{X}) : \nabla_{\mathbf{X}}(\delta\tilde{\mathbf{u}}) \ \mathrm{d}\Omega_0 = 0 \tag{25}$$

$$\mathbf{R}^p = \int_{\Omega_0} (\mathbf{F}^p_{n+1} - \mathbf{F}^p_n - \Delta t (\mathbf{F}^e)^{-1} \cdot \dot{\bar{\varepsilon}}^p \mathbf{N} \cdot \mathbf{F}) : \delta\mathbf{F}^p \ \mathrm{d}\Omega_0 = 0 \tag{26}$$

$$\mathbf{R}^{\lambda_m} = -\left(\frac{1}{|\Omega_0|} \int_{\Omega_0} \mathbf{P}(\mathbf{F}, \mathbf{F}^p, \theta, \mathbf{X}) \mathrm{d}\Omega_0 - \overline{\mathbf{P}} \right) : \delta\boldsymbol{\lambda}_m = 0 \ . \tag{27}$$

From an electrical viewpoint, we solve the electrically stationary continuity equation in terms of electric current density:

$$\mathbf{R}^e = -\int_{\Omega_0} \mathbb{J} \cdot \nabla_{\mathbf{X}}(\delta\tilde{\phi}) \ \mathrm{d}\Omega_0 = 0 \ . \tag{28}$$

These four equations are solved in a finite element mesh consisting of a representation of the composite mesostructure. Macroscopic values of the deformation and electric field are imposed to solve the boundary value problem under periodic boundary conditions[2]. A Newton-Raphson non-linear solver is employed to achieve the solution with a relative tolerance of 10^{-5}. All the equations were implemented in the FEniCs python module and, therefore, the derivatives are symbolically calculated by the module. The resulting non-linear system of equations is solved implicitly via monolithic scheme, accounting for every Jacobian submatrix, which reads as:

$$\begin{bmatrix} \mathbf{K}_{uu} & \mathbf{K}_{up} & \mathbf{K}_{u\lambda_m} & \mathbf{0} \\ \mathbf{K}_{pu} & \mathbf{K}_{pp} & \mathbf{K}_{p\lambda_m} & \mathbf{0} \\ \mathbf{K}_{\lambda_m u} & \mathbf{K}_{\lambda_m p} & \mathbf{K}_{\lambda_m \lambda_m} & \mathbf{0} \\ \mathbf{K}_{\phi u} & \mathbf{0} & \mathbf{K}_{\phi\lambda_m} & \mathbf{K}_{\phi\phi} \end{bmatrix} \begin{Bmatrix} \Delta u \\ \Delta F_p \\ \Delta\lambda_m \\ \Delta\phi \end{Bmatrix} = - \begin{Bmatrix} \mathbf{R}^u \\ \mathbf{R}^p \\ \mathbf{R}^{\lambda_m} \\ \mathbf{R}^e \end{Bmatrix} \tag{29}$$

where $\mathbf{K}_{xy}$ represents the derivative of the residual $\mathbf{R}^x$ with respect to the variable y.

Macroscopic formulation

In this section, we propose an orthotropic formulation for the thermo-electro-mechanical response of 3D printed PLA/CB composite at a macroscopic scale. Due to the diverse printing orientations considered, this framework will make use of rotation tensors to work in a reference orientation configuration, i.e., longitudinal orientation. As for the homogenised approach, this formulation is set considering finite deformations.

[2] To study the thermal effect on the mechanical or electrical contributions, a temperature can be prescribed in the whole RVE. Yet, it is not considered as a field variable in the boundary value problem.

Orthotropic mechanical behaviour of printed PLA/CB

The mechanical constitutive behaviour of the composite was modelled with an orthotropic thermo-elasto-viscoplastic rheological model [3]. As for the homogenised approach, the rheological model consists of a unique branch with a hyperelastic spring in series with a viscoplastic dashpot. The considered energy strain function is based on an orthotropic Saint Venant-Kirchhoff model [3, 4]:

$$\psi(\mathbf{C}^{\mathrm{e}}) = \frac{1}{2}\sum_{i,j}^{3} \lambda_{ij}(\theta)\mathrm{tr}(\mathbf{E}^{\mathrm{e}} \cdot \mathbf{L}_{ii})\mathrm{tr}(\mathbf{E}^{\mathrm{e}} \cdot \mathbf{L}_{jj}) + \sum_{i,j\neq i}^{3} \mu_{ij}(\theta)\mathrm{tr}(\mathbf{E}^{\mathrm{e}} \cdot \mathbf{L}_{ii} \cdot \mathbf{E}^{\mathrm{e}} \cdot \mathbf{L}_{jj}), \tag{30}$$

where $\mathbf{E}^{\mathrm{e}} = \frac{1}{2}(\mathbf{C}^{\mathrm{e}} - \mathbf{I})$ is the elastic Green-Lagrange strain tensor, λ_{ij} are the Lame's constants in each direction, μ_{ij} are the shear moduli in each direction, and $\mathbf{L}_{ii}$ are the so called structural tensors that allow to define principal directions within the model. To obtain the structural tensors related to the principal material directions, we consider a set of orthogonal unit base vectors coinciding with the desired directions:

$$\mathbf{L}_{ii} = \mathbf{l}_i \otimes \mathbf{l}_i \quad i \in \{1, 2, 3\} \tag{31}$$

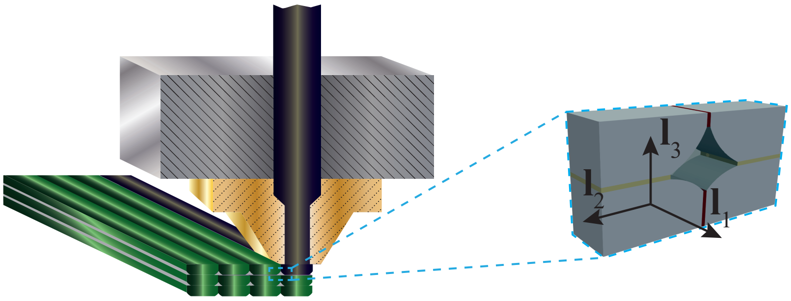

Supplementary Figure 2. Diagram of the orthogonal unit base vectors of the reference orientation configuration $\hat{\Omega}$, i.e., longitudinal ($\mathbf{l}_1$).

The Second Piola-Kirchhoff stress can be obtained by deriving the strain energy function with respect to the elastic Green-Lagrange strain tensor as:

$$\overline{\mathbf{S}} = \frac{\partial \psi}{\partial \mathbf{E}^{\mathrm{e}}} = \sum_{i,j}^{3} \lambda_{ij}(\theta)\mathrm{tr}(\mathbf{E}^{\mathrm{e}} \cdot \mathbf{L}_{jj})\mathbf{L}_{ii} + 2\sum_{i,j\neq i}^{3} \mu_{ij}(\theta)\mathbf{L}_{ii} \cdot \mathbf{E}^{\mathrm{e}} \cdot \mathbf{L}_{jj} \tag{32}$$

The Piola stress can be alternatively obtained as:

$$\mathbf{P} = \mathbf{F} \cdot (\mathbf{F}^{\mathrm{p}})^{-1} \cdot \overline{\mathbf{S}} \cdot ((\mathbf{F}^{\mathrm{p}})^{-1})^{T} = \mathbf{F}^{\mathrm{e}} \cdot \left(\sum_{i,j}^{3} \lambda_{ij}(\theta)\mathrm{tr}(\mathbf{E}^{\mathrm{e}} \cdot \mathbf{L}_{jj})\mathbf{L}_{ii} + 2\sum_{i,j\neq i}^{3} \mu_{ij}(\theta)\mathbf{L}_{ii} \cdot \mathbf{E}^{\mathrm{e}} \cdot \mathbf{L}_{jj} \right) \cdot ((\mathbf{F}^{\mathrm{p}})^{-1})^{T}, \tag{33}$$

To allow the use of this formulation for any printing orientation, rotation tensors ($\mathbf{Q}$) were added to transform current variables to the reference orientation configuration ($\hat{\Omega}$), that is set as the longitudinal printing direction. The proposed algorithm, which calculates the Piola Kirchhoff stress at the current orientation configuration (Ω), consists in [5]:

1. Transform the elastic Green-Lagrange strain from the current orientation configuration to the reference orientation configuration.
$$\hat{\mathbf{E}}^{\mathrm{e}} = \mathbf{Q}^{\mathrm{T}} \cdot \mathbf{E}^{\mathrm{e}} \cdot \mathbf{Q} \ . \tag{34}$$

2. Compute the Second Piola-Kirchhoff stress at the reference orientation configuration.
$$\hat{\mathbf{S}} = \sum_{i,j}^{3} \lambda_{ij}(\theta)\mathrm{tr}(\hat{\mathbf{E}}^{\mathrm{e}} \cdot \mathbf{L}_{jj})\mathbf{L}_{ii} + 2\sum_{i,j\neq i}^{3} \mu_{ij}(\theta)\mathbf{L}_{ii} \cdot \hat{\mathbf{E}}^{\mathrm{e}} \cdot \mathbf{L}_{jj} \ . \tag{35}$$

3. Transform the Second Piola-Kirchhoff stress to the current orientation configuration.
$$\mathbf{S} = \mathbf{Q} \cdot \hat{\mathbf{S}} \cdot \mathbf{Q}^{\mathrm{T}} \ . \tag{36}$$

The rotation matrix used in the previous expressions is defined as the product of the rotation matrix along each coordinated axis in the reference orientation configuration:

$$\mathbf{Q} = \mathbf{Q}_{\hat{\mathbf{X}}} \cdot \mathbf{Q}_{\hat{\mathbf{Y}}} \cdot \mathbf{Q}_{\hat{\mathbf{Z}}} \tag{37}$$

Where each rotation tensor reads as:

$$\mathbf{Q}_{\hat{\mathbf{X}}} = \begin{bmatrix} 1 & 0 & 0 \\ 0 & cos(\varphi_x) & -sen(\varphi_x) \\ 0 & sen(\varphi_x) & cos(\varphi_x) \end{bmatrix}; \mathbf{Q}_{\hat{\mathbf{Y}}} = \begin{bmatrix} cos(\varphi_y) & 0 & sen(\varphi_y) \\ 0 & 1 & 0 \\ -sen(\varphi_y) & 0 & cos(\varphi_y) \end{bmatrix}; \mathbf{Q}_{\hat{\mathbf{Z}}} = \begin{bmatrix} cos(\varphi_z) & sen(\varphi_z) & 0 \\ -sen(\varphi_z) & cos(\varphi_z) & 0 \\ 0 & 0 & 1 \end{bmatrix}, \tag{38}$$

where φ_x, φ_y and φ_z are the rotation angles with respect to $\mathbf{l}_1$, $\mathbf{l}_2$ and $\mathbf{l}_3$ respectively.
To obtain the plastic deformation, $\mathbf{F}^{\mathrm{p}}$ is calculated following the same methodology as the one presented in the homogenisation formulation.

Orthotropic electrical behaviour of printed PLA/CB

Due to the imperfect adhesion between filaments during the printing process, the electrical response of conductive PLA/CB printed pieces is also orthotropic. As mentioned in the previous section, the reference orientation configuration was the longitudinal one, considering as principal directions: $\mathbf{l}_1$, the direction along filament; $\mathbf{l}_2$, the direction between adjacent filaments; $\mathbf{l}_3$, building direction. The conductivity tensor is defined as follows:

$$\hat{\mathbf{\Sigma}} = \begin{bmatrix} \hat{\Sigma}_{11} & 0 & 0 \\ 0 & \hat{\Sigma}_{22} & 0 \\ 0 & 0 & \hat{\Sigma}_{33} \end{bmatrix}. \tag{39}$$

As observed in compressive mechano-electrical experimental results (see section: Additional mechano-electrical tests), there is a maximum conductivity that this material can reach at each direction (Σ_{ii}^{crit}). In addition, when the particles at the microscale are sufficiently separated to stop tunnelling effect, there is a cease of conductive properties, i.e., $\Sigma = 0$ S/m. As the conductive properties vary with strain between these two values, we proposed sigmoid-based functions to describe the conductivity variation at each principal direction. Following this experimental evidence, the conductivity along $\mathbf{l}_1$ (i.e., filament direction) is proposed dependent on the square root of the fourth invariant of the right Cauchy-Green tensor along longitudinal direction ($\sqrt{I_4^1}$) as well as with the constriction deformation (dA/dA_o)[6]. The former variable determines the deformation along the longitudinal direction, projecting the right Cauchy-Green tensor ($\mathbf{C} = \mathbf{F}^{\mathrm{T}} \cdot \mathbf{F}$) with the longitudinal structural tensor ($\mathbf{L}_{11} = \mathbf{l}_1 \otimes \mathbf{l}_1$); $I_4^l = \mathbf{C} : \mathbf{L}_{11}$. The latter variable determines the deformation caused by Poisson's effect in the directions perpendicular to the longitudinal one; $dA/dA_o = J_F \sqrt{(\mathbf{F}^{-1})^{\mathrm{T}} \cdot \mathbf{l}_1 \cdot (\mathbf{F}^{-1})^{\mathrm{T}} \cdot \mathbf{l}_1}$. This conductivity is set as

$$\hat{\Sigma}_{11} = \frac{a_{11}(\Sigma_{11}^{crit}, \Sigma_{11}^{\theta})}{b_{11}(\Sigma_{11}^{crit}, \Sigma_{11}^{\theta}) + exp(C_{11}^F(\sqrt{I_4^1} - 1) - C_{dA}^F(1 - dA/dA_o))}, \tag{40}$$

where C_{11}^F is the sensitivity parameter related to the longitudinal deformation and C_{dA}^F is the sensitivity parameter related to the constriction deformation.
In the case of the remaining principal directions $\mathbf{l}_2$ (i.e., adjacent filaments or transverse) and $\mathbf{l}_3$ (i.e., building direction or vertical), their conductivity is set to be dependent with the fourth invariant of the right Cauchy-Green tensor along the studied direction as:

$$\hat{\Sigma}_{ii} = \frac{a_{ii}(\Sigma_{ii}^{crit}, \Sigma_{11}^{\theta})}{b_{ii}(\Sigma_{ii}^{crit}, \Sigma_{ii}^{\theta}) + exp(C_{ii}^F(\sqrt{I_4^i} - 1))} \quad \forall i \in \{2, 3\}, \tag{41}$$

where C_{ii}^F is the sensitivity parameter related to the ith deformation. As in Eq. 40, the variables $a_{ii} = \Sigma_{ii}^{\theta}\Sigma_{ii}^{crit}/(\Sigma_{ii}^{crit} - \Sigma_{ii}^{\theta})$ and $b_{ii} = \Sigma_{ii}^{\theta}/(\Sigma_{ii}^{crit} - \Sigma_{ii}^{\theta})$ define the conductivity without any deformation (Σ_{ii}^{θ}) as well as the maximum conductivity allowed for each direction (Σ_{ii}^{crit}).
Note that along the formulation of the different principal directions conductivities, the variable Σ_{ii}^{θ} has been used. This variable captures the conductivity dependence with temperature, and it is set as:

$$\Sigma_{ii}^{\theta} = \Sigma_{ii}^{ref}(1 - \alpha_T(\theta - \theta^{ref})), \tag{42}$$

where Σ_{ii}^{ref} is the conductivity at i^{th} direction at the reference temperature (25°C).
To transform the conductivity matrix to the chosen printing orientation, the rotation tensor was used:

$$\mathbf{\Sigma} = \mathbf{Q}^{\mathrm{T}} \cdot \hat{\mathbf{\Sigma}} \cdot \mathbf{Q} \tag{43}$$

Thermal behaviour of printed PLA/CB

Although the electric and mechanical behaviours were defined orthotropic, the thermal behaviour is considered isotropic. This decision is motivated by the homogeneous thermal response observed in the electro-thermal and thermo-mehano-electrical experiments. The thermal conductivity and specific heat were assumed independent of temperature due to the low-temperature range considered in this work. Regarding the thermal dependencies of the mechanical parameters, we proposed a decrease of Young's modulus, yield stress and hardening as stated in Eq. 44:

$$\sigma_{\mathrm{Y}}(\theta) = \sigma_{\mathrm{Y}}^{\mathrm{ref}} - C_{\mathrm{Y}}(\theta - \theta^{\mathrm{ref}})^2 \ ; \ E_i(\theta) = E_i^{\mathrm{ref}} - C_E(\theta - \theta^{\mathrm{ref}})^2 \ ; \ H(\theta) = H^{\mathrm{ref}} + C_{\mathrm{H}}(\theta - \theta^{\mathrm{ref}}) \tag{44}$$

where C_θ, C_{Y} and C_{H} are constants that represent the decrease of stiffness, yield stress and hardening with temperature respectively, whereas E_i^{ref}, $\sigma_{\mathrm{Y}}^{\mathrm{ref}}$ and H^{ref} represent the values at the reference temperature.

Thermodynamics

The Helmholtz free energy is dependent on the deformation gradient, $\mathbf{F}$, the electric field, $\mathbb{E}$, the absolute temperature, θ, and the plastic deformation gradient, $\mathbf{F}^{\mathrm{p}}$ (disregarding the hardening), as

$$\psi(\mathbf{F}, \mathbb{E}, \theta, \mathbf{F}^{\mathrm{p}}) = e - \mathbb{E} \cdot \mathbb{D} - \theta\eta \ , \tag{45}$$

where η is the entropy per unit volume, e is the internal energy per unit volume and $\mathbb{D}$ is the electric displacement.
From the first law of thermodynamics, the internal energy is obtained as

$$\dot{e} = \mathbf{P} : \dot{\mathbf{F}} + \mathbb{E} \cdot \dot{\mathbb{D}} + \mathbb{E} \cdot \mathbb{J} + R - \nabla_{\mathbf{X}} \cdot \mathbf{Q} \quad , \tag{46}$$

where R is the heat resource, $\mathbf{Q}$ is the heat flux, $\mathbb{J}$ is the electrical current density and $\mathbf{P}$ is the first Piola-Kirchhoff stress tensor. The second law of thermodynamics reads

$$\dot{\eta} + \nabla_{\mathbf{X}} \cdot (\frac{\mathbf{Q}}{\theta}) - \frac{R}{\theta} \geq 0 \ . \tag{47}$$

Combining both laws, Eqs. 46 and 47, it is possible to write the Clausius-Duhem inequality in terms of the internal energy.

$$\mathcal{D}_e = \mathbf{P} : \dot{\mathbf{F}} + \mathbb{E} \cdot \dot{\mathbb{D}} + \theta\dot{\eta} + \mathbb{E} \cdot \mathbb{J} - \dot{e} \geq 0 \ . \tag{48}$$

Accounting for the rate of the Helmholtz free energy

$$\dot{\psi} = \partial_{\mathbf{F}}\psi : \dot{\mathbf{F}} + \partial_{\mathbb{E}}\psi \cdot \dot{\mathbb{E}} + \partial_\theta\psi\dot{\theta} + \partial_{\mathbf{F}^{\mathrm{p}}}\psi : \dot{\mathbf{F}}^{\mathrm{p}} = \dot{e} - \dot{\mathbb{E}} \cdot \mathbb{D} - \mathbb{E} \cdot \dot{\mathbb{D}} - \dot{\theta}\eta - \theta\dot{\eta} \ , \tag{49}$$

it is possible to substitute the internal energy from in Eq. 48. Thus, the Clausius-Duhem inequality in terms of the free energy reads:

$$\mathcal{D}_\psi = (\mathbf{P} - \partial_{\mathbf{F}}\psi) : \dot{\mathbf{F}} - (\mathbb{D} + \partial_{\mathbb{E}}\psi) \cdot \dot{\mathbb{E}} - (\eta + \partial_\theta\psi)\dot{\theta} + \mathbb{E} \cdot \mathbb{J} - \partial_{\mathbf{F}^{\mathrm{p}}}\psi : \dot{\mathbf{F}}^{\mathrm{p}} \geq 0 \tag{50}$$

By fulfilling the inequality it is possible to obtain the following constitutive relations:

$$\mathbf{P} = \partial_{\mathbf{F}}\psi \ , \quad \mathbb{D} = -\partial_{\mathbb{E}}\psi \ , \quad \eta = -\partial_\theta\psi \ , \tag{51}$$

leaving a reduced inequality:

$$\mathcal{D}_\psi = \mathbb{E} \cdot \mathbb{J} - \partial_{\mathbf{F}^{\mathrm{p}}}\psi : \dot{\mathbf{F}}^{\mathrm{p}} \geq 0 \ . \tag{52}$$

The first term, referred to the Joule heating, always fulfil the condition, due to the definition of the electric current density, $\mathbb{J} = \Sigma J_F \mathbf{F}^{-1} \cdot \mathbf{F}^{-T} \cdot \mathbb{E}$, where the electrical conductivity is always positive. Regarding the plastic deformation term, by considering an associative flow rule the inequality is fulfilled, note that thermodynamic consistency is also ensured in the case of isotropic hardening.

Governing equations

From a macroscopic mechanical perspective, the linear momentum balance is solved in the reference configuration.

$$\nabla_{\mathbf{X}} \cdot \mathbf{P} = \mathbf{0}, \tag{53}$$

The plastic deformation component has been set as a degree of freedom and is computed by solving the time variation of the plastic deformation gradient, see Eq. 8.
Analogously with the mesoscopic electrical contribution, the electric potential field ϕ can be obtained by solving the continuity equation.

$$\nabla_{\mathbf{X}} \cdot \mathbb{J} = 0 \tag{54}$$

For the thermal governing equation, the transient heat balance is set as

$$\varrho\dot{\theta} - \nabla_{\mathbf{X}} \cdot (\kappa J_F \mathbf{F}^{-1} \cdot \mathbf{F}^{-T} \cdot \nabla_{\mathbf{X}}\theta) - \mathbb{J} \cdot \mathbb{E} + h(\theta - \theta^{\text{ref}}) = 0 \ , \tag{55}$$

where ϱ is the volumetric heat capacity (density in the reference configuration multiplied by heat capacity), k is the thermal conductivity, h is the convection coefficient and $\mathbb{J} \cdot \mathbb{E}$ is the volumetric heat generation caused by Joule effect.
Gathering the previous strong form formulations, we can summarise the residual expressions used to solve the coupled non-linear system of equations in our finite element model. From a mechanical perspective, we account for solving the mechanical equilibrium, Eq. 56, and the residual of the plastic deformation gradient (as we consider it as an independent variable):

$$\mathbf{R}^{\text{m}} = \int_{\Omega_0} \mathbf{P}(\mathbf{F}, \mathbf{F}^{\text{p}}, \theta, \mathbf{X}) : \nabla_{\mathbf{X}}(\delta\mathbf{u}) \ \text{d}\Omega_0 = 0 \tag{56}$$

$$\mathbf{R}^{\text{p}} = \int_{\Omega_0} (\mathbf{F}^{\text{p}}_{n+1} - \mathbf{F}^{\text{p}}_{n} - \Delta t(\mathbf{F}^{e})^{-1} \cdot \dot{\bar{\varepsilon}}^{\text{p}}\mathbf{N} \cdot \mathbf{F}) : \delta\mathbf{F}^{\text{p}} \ \text{d}\Omega_0 = 0 \tag{57}$$

From an electrical viewpoint, we solve the electrically stationary continuity equation in terms of electric current density:

$$\mathbf{R}^{\text{e}} = -\int_{\Omega_0} \mathbb{J} \cdot \nabla_{\mathbf{X}}(\delta\phi) \ \text{d}\Omega_0 = 0. \tag{58}$$

To obtain the solution of the temperature field, we solve the transient heat balance, Eq. 59:

$$\mathbf{R}^{th} = \int_{\Omega_0} (\varrho \frac{\theta - \theta_t}{dt})\delta\theta + (\kappa J_F \mathbf{F}^{-1} \cdot \mathbf{F}^{-T} \cdot \nabla_{\mathbf{X}}\theta) \cdot \nabla_{\mathbf{X}}(\delta\theta) - (\mathbb{J} \cdot \mathbb{E})\delta\theta \ \text{d}\Omega_0 + \int_{\Gamma_0} h(\theta - \theta^{\text{ref}})\delta\theta \ \text{d}\Gamma_0 = 0 \tag{59}$$

Computational details of the optimisation algorithm

The Particle Swarm Optimisation algorithm used a velocity vector to evaluate possible optimal solutions. This vector is determined by three components: i) a momentum component; ii) a cognitive component, which influences the ith particle to search solutions near the best solution found so far by a specific particle ($\mathbf{PBEST}_i^j$); and iii) a social component, which leads the ith particle to search solutions near the best solution found so far by all the particles ($\mathbf{GBEST}^j$). Thus, the velocity of the ith particle at the jth iteration is set as:

$$\mathbf{V}_i^j = \mathbf{V}_{mom,i}^j + \mathbf{V}_{cog,i}^j + \mathbf{V}_{soc,i}^j \ . \tag{60}$$

The momentum component decreases its importance over each iteration. This allows for a more precise search near the best solutions during the last iterations, while performing a more general search during the first iterations. This component reads as:

$$\mathbf{V}_{mom,i}^j = \left(\left(1 - \frac{j}{n}\right)(w_{max} - w_{min}) + w_{min} \right) \mathbf{V}_i^{j-1}, \tag{61}$$

where n is the maximum number of iterations, w_{max} is a parameter that defines the maximum momentum and w_{min} is a parameter that defines the minimum momentum.
The cognitive component allows for a specific particle to search for solutions near the best solution found by that specific particle. This solution is known as personal best ($\mathbf{PBEST}_i^j$). The expression is set as:

$$\mathbf{V}_{cog,i}^j = c_1 \mathbf{R} \cdot (\mathbf{PBEST}_i^j - \mathbf{X}_i^{j-1}), \tag{62}$$

where c_1 is a weight parameter for the cognitive component, $\mathbf{R}$ is a random vector which components absolute values are smaller than 1, and $\mathbf{X}_i^{j-1}$ is the solution of the ith particle at the former iteration. The social component allows for each particle to search for solutions near the best solution found by the set of particles. This solution is known as global best ($\mathbf{GBEST}^j$). The expression is set as:

$$\mathbf{V}_{soc,i}^j = c_2\mathbf{R} \cdot (\mathbf{GBEST}^j - \mathbf{X}_i^{j-1}), \tag{63}$$

where c_2 is a weight parameter for the social component. Thus, the next position of the ith particle within the solution space to evaluate the objective function is defined as:

$$\mathbf{X}_i^j = \mathbf{X}_i^{j-1} + \mathbf{V}_i^j. \tag{64}$$

Both $\mathbf{PBEST}_i^j$ and $\mathbf{GBEST}^j$ are changed over iterations (j) when their evaluation in the objective function is more optimal than the ones obtained before. Every optimisation problem employed an objective function defined as the root mean squared error (RMSE) between the experimental and model data. Thus, an optimal solution consists of the minimisation of the objective function:

$$f(\mathbf{X}_i^j) < f(\mathbf{PBEST}_i^j) \implies \mathbf{PBEST}_i^{j+1} = X_i^j \quad \forall\{i,j\} \tag{65}$$

$$f(\mathbf{X}_i^j) < f(\mathbf{GBEST}^j) \implies \mathbf{GBEST}^{j+1} = X_i^j \quad \forall\{i,j\}\ . \tag{66}$$

Isotropic model for mechano-electrical response of conductive filament at the macroscale

As observed in the mechano-electrical tests at a previous work [2], the resistivity variation is only linear during the elastic regime. Once plasticity is reached, this tendency slightly reduces during the initial stages, to increase again in a linear way after that stretch values ($\lambda \approx 1.025$). To capture this macroscopic phenomena, we have proposed a relation between conductivity and the first invariant of the deformation gradient, $I_1^F = tr(\mathbf{F})$. This proposal is set based on the microstructure of PLA/CB composite. The microscopic CB particles do not form perfectly aligned paths, but they constitute a randomly dispersed net of particles. Thus, the composite conductivity will not only depend on the deformation along the electric field direction, but on the other two directions as well. To account for all the principal directions deformations, we have made use of I_1^F:

$$\Sigma(\mathbf{F}) = \Sigma_{ref}(1 - C_F(I_1^F - 3)), \tag{67}$$

where Σ_{ref} is the conductivity at the undeformed state and C_F is the strain-sensitivity parameter.

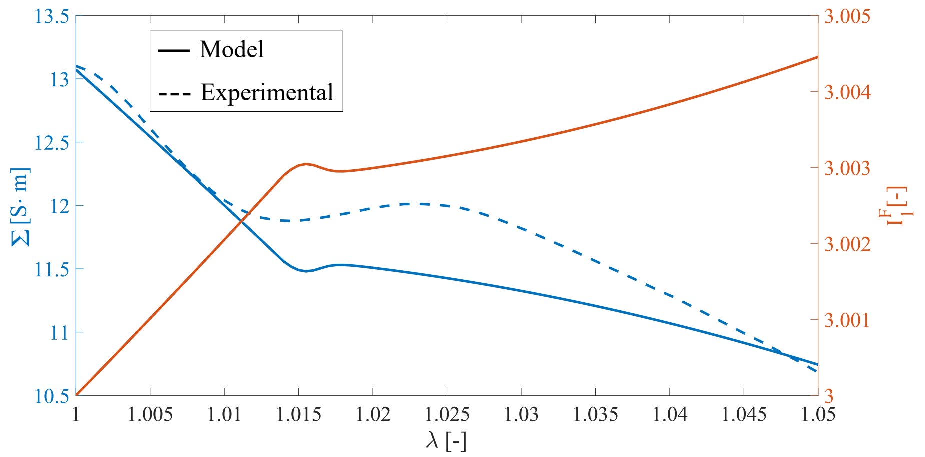

Supplementary Figure 3. Relation between the first invariant of the deformation gradient and the mechano-electrical response of PLA/CB. In the left y-axis the conductivity during a mechano-electrical test of PLA/CB filament (experiment) and the conductivity results following the proposed numerical model are plotted versus the mechanical stretch. In the right y-axis, the evolution of the first invariant is represented. Experimental data obtained from [2]

Due to the deviatoric nature of the plastic regime, once plasticity is reached the transverse components of the deformation gradient decrease faster than during the elastic regime. Therefore, the I_1^F is slightly

reduced at this stage. In Supplementary Fig. 3 left y-axis, the average variation of conductivity during the mechano-electrical testing at the filament scale is presented along with the conductivity obtained by applying the proposed model. The variation of I_1^F is represented in the right y-axis. It can be noticed how at λ values near 1.015, where plasticity is reached in the elasto-plastic model a reduction in tendency is found.

Identification of macroscopic elastic parameters via homogenisation

Unlike isotropic materials, orthotropic elastic properties cannot be fully related to each other (e.g., for an isotropic material the shear modulus can be obtained if the Poisson's ratio and the Young's modulus are known). In the proposed orthotropic hyperelastic model [3, 4], the orthotropic set of Lamé parameters are needed, i.e., Lamé's first parameters ($\lambda_{ij} \quad \forall i, j \in \{1, 2, 3\}$) and shear moduli ($\mu_{ij} \quad \forall i \neq j \in 1, 2, 3$).

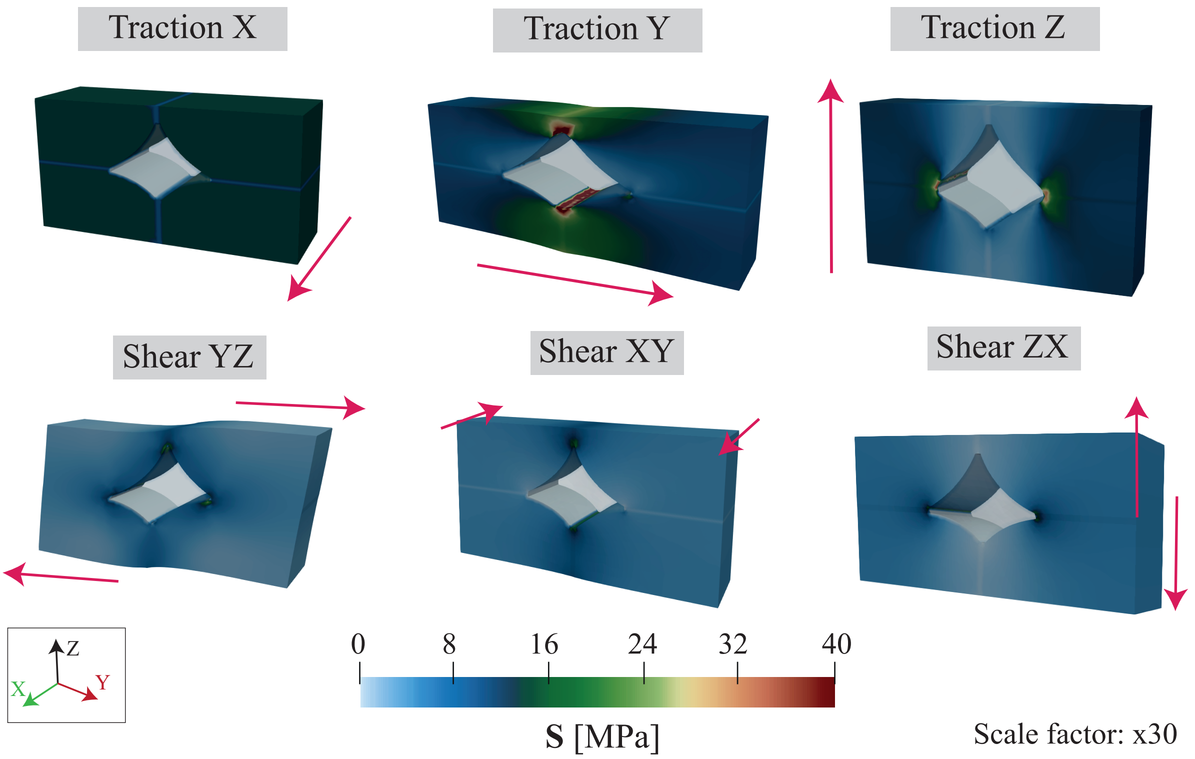

Supplementary Figure 4. Stress results of each deformation state. In the first row every uniaxial tensile simulation is presented. In the second row every pure shear simulation is presented. A macroscopic stretch value of 0.005 is applied for every condition.

The former set of parameters can be obtained by knowing the principal directions Young's moduli (i.e., along longitudinal, transverse and vertical directions) as well as knowing the Poisson's ratios:

$$\lambda_{ii} = E_i \frac{1 - \nu_{jk}\nu_{kj}}{\Delta}, \tag{68}$$

$$\lambda_{ij} = \lambda_{ji} = E_i \frac{\nu_{ij} + \nu_{kj}\nu_{ik}}{\Delta} \quad (k \neq j \neq i, \quad \forall i, j \in \{1, 2, 3\}) \tag{69}$$

where:

$$\Delta = 1 - \nu_{12}\nu_{21} - \nu_{23}\nu_{32} - \nu_{31}\nu_{13} - 2\nu_{21}\nu_{32}\nu_{13}. \tag{70}$$

It is important to remark that, in case of orthotropic materials, there is a relation between ν_{ij} and ν_{ji}, based on the occupied Young's moduli:

$$\nu_{ij} = \nu_{ji} \frac{E_j}{E_i} \tag{71}$$

To obtain the Young's moduli and Poisson's ratios, uniaxial tensile simulations were performed at the mesoscale to calculate each parameter based on the average of the desired solution fields. E_i is calculated

as the slope between the macroscopic Second Piola-Kirchhoff stress at the ith direction ($\overline{\mathbf{S}}_{ii}$) and the macroscopic Green-Lagrange strain at the ith direction ($\overline{\mathbf{E}}_{ii}$). To be consistent with the Hill-Mandel or macro-homogeneity condition [7, 8], $\overline{\mathbf{S}}_{ii}$ and $\overline{\mathbf{E}}_{ii}$ were computed from the averaged deformation gradient ($\overline{\mathbf{F}}_{ij} = \frac{1}{\Omega} \int_{\Omega_o} \boldsymbol{F}_{ij} d\Omega$) and the first Piola-Kirchhoff stress ($\overline{\mathbf{P}}_{ij} = \frac{1}{\Omega} \int_{\Omega_o} \boldsymbol{P}_{ij} d\Omega$). ν_{ji} is calculated as the slope between the average of Green-Lagrange strain in the jth direction with respect to the ith one, where the uniaxial loading is applied.
In the case of the shear moduli obtention, pure shear conditions on each principal plane were applied to RVEs. Then, they were calculated as the slope between the average of the ij^{th} component of the Second Piola-Kirchhoff stress tensor with respect to the desired tangential component of the average of Green-Lagrange strain tensor.

Macroscopic simulations of the electro-thermal response of PLA/CB

In this section the macroscopic simulation results for electro-thermal tests using electric fields of 125 V/m and 187.5 V/m are represented against the experimental data in Supplementary Fig. 5, complementing the experimental results presented in Fig. 3.c where an electric field of 250 V/m was applied. It can be seen how the model captures the difference in heating depending on the printing orientation, as well as the resistivity variation.

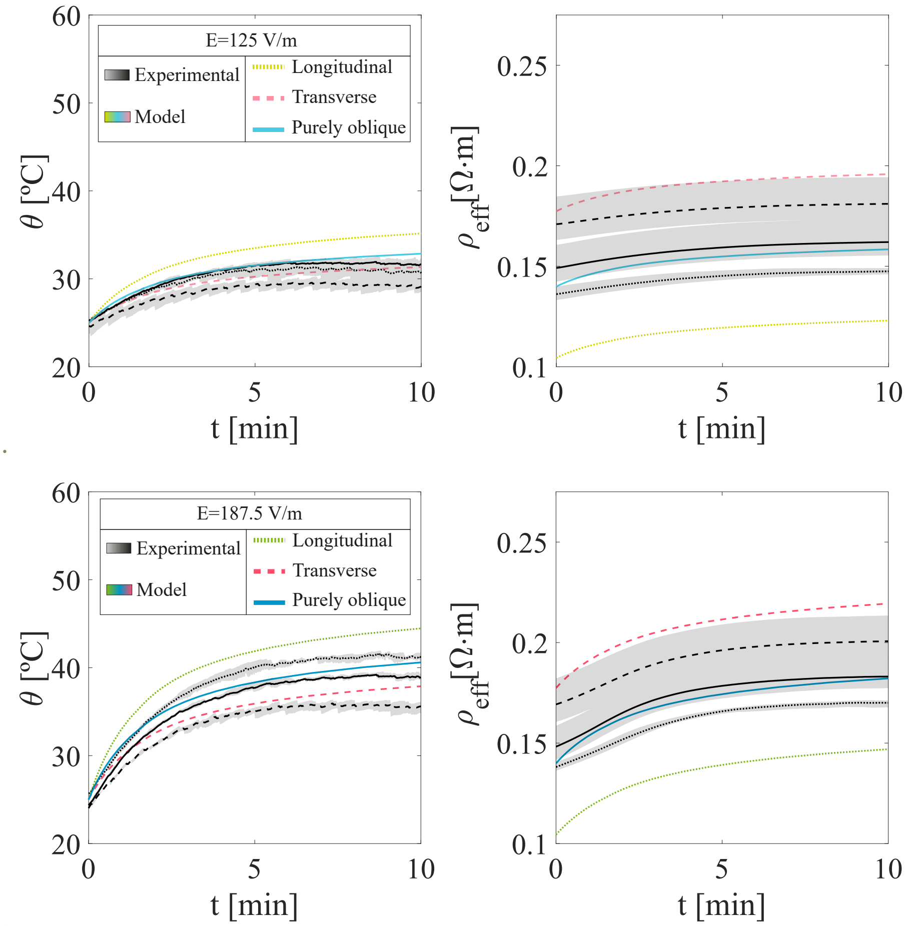

Supplementary Figure 5. Electro-thermal simulation results for electric fields of 125 and 187.5 V/m. The experimental results of wide rectangular samples employing gripping electrodes are displayed in grey. The solid/dashed lines represent the average values of the response, whereas the shaded areas represent the experimental deviation. Three samples were considered for each experimental condition. Only one simulation was used for each condition, feeding the model with the average parameters calibrated via PSO algorithm.

Macroscopic simulations of the thermo-mechanical response of PLA/CB

In this section the macroscopic simulation results for thermo-mechanical tests imposing temperatures of 25 and 35 °C are represented against the experimental data in Supplementary Fig. 6, complementing the experimental results presented in Fig. 3.a at 45 °C. The experimental results are obtained from wide rectangular section samples.

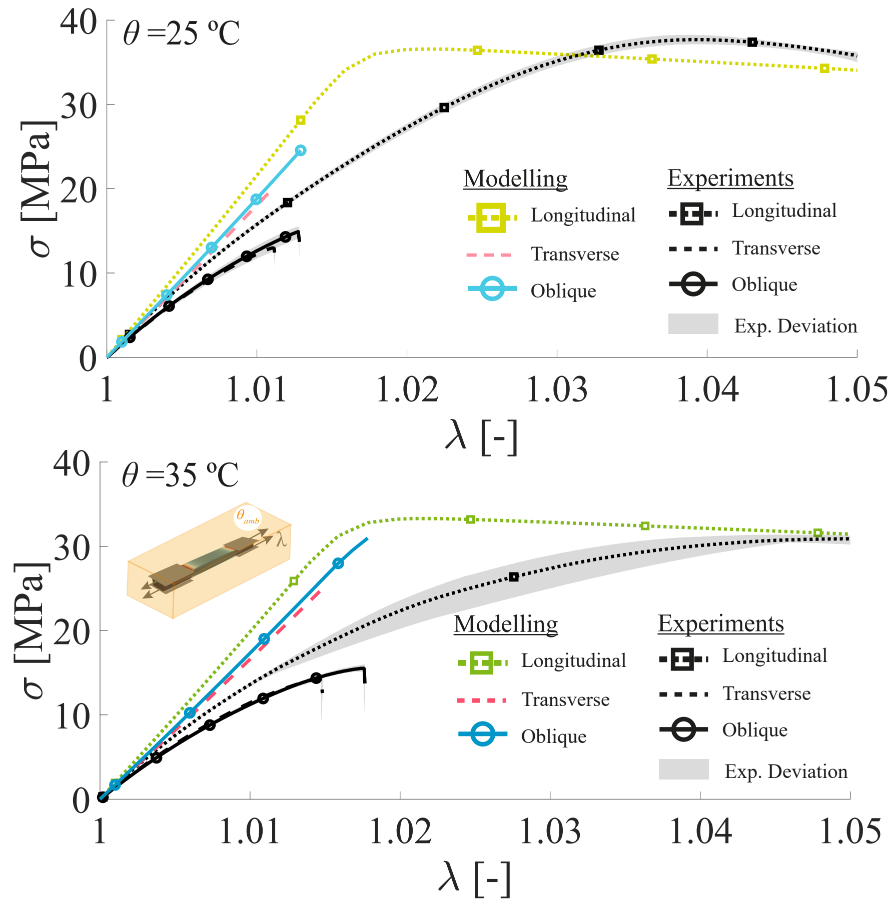

Supplementary Figure 6. **Thermo-mechanical simulation results for temperatures of 25 and 35 °C.** The experimental results of wide rectangular samples are displayed in grey. The solid/dashed lines represent the average values of the response, whereas the shaded areas represent the experimental deviation. Three samples were considered for each experimental condition. Only one simulation was used for each condition, feeding the model with the average parameters obtained from homogenisation.

Calibrated parameters for the simulations

In this section every parameter used for solving both the mesoscopic and macroscopic problems is displayed.

Mesoscopic problem

The elasto-viscoplastic parameters used in the homogenisation framework are presented in Supplementary Table 1 dividing them into the four phases considered. The electrical and mechano-electrical parameters are presented in Supplementary Table 2.

Both Young's modulus and electrical conductivity at the reference temperature of the pure filament were not calibrated via optimisation approaches. These values were obtained directly from experimental data. The Young's modulus was obtained from the stress-strain experimental curves of extruded filaments, whereas the filament conductivity was measured from filaments extruded and deposited in the printing bed.

Supplementary Table 1: Elasto-viscoplastic parameters considered at the mesoscale for every phase in the RVEs.

	Mechanical parameters								
Phases	E [MPa]	ν [-]	$\sigma_{\mathrm{Y}}^{\mathrm{ref}}$ [MPa]	$\dot{\varepsilon}_{\mathrm{o}}[s^{-1}]$	C [-]	H^{ref}	σ_s [MPa]	γ [-]	η [-]
Filament	2300	0.4	35	1 e-4	0.06	0.05	$\sigma_Y(\theta)/1.15$	-	-
Inter-layer	Eq. 10	0.4	Eq. 9	1 e-4	0.06	0.05	$\sigma_Y(\theta)/1.15$	0.3	-
Inter-filament	Eq. 10	0.4	Eq. 9	1 e-4	0.06	0.05	$\sigma_Y(\theta)/1.15$	-	0.3
Void	1e-10	0.2	-	-	-	-	-	-	-

Supplementary Table 2: Electrical and mechano-electrical parameters considered at the mesoscale for every phase in the RVEs.

	Electro and mechano-electrical parameters			
Phases	Σ $[\frac{S}{m}]$	C_F [-]	α [-]	β
Filament	11.76	30	-	-
Inter-layer	Eq. 13	30	0.095	-
Inter-filament	Eq. 13	30	-	0.096
Void	1e-12	-	-	-

Macroscopic problem

The orthotropic elasto-viscoplastic parameters obtained from the optimisation procedure and used for macroscopic simulations in the macroscopic simulations are presented in Supplementary Table 3. The electrical and mechano-electrical parameters are presented in Supplementary Table 4. The thermal, thermo-electrical and thermo-mechanical parameters are presented in Supplementary Table 5.

Supplementary Table 3: Orthotropic elasto-viscoplastic parameters considered at the macroscopic scale and obtained from the optimisation procedure.

E_1 [MPa]	E_2 [MPa]	E_3 [MPa]	μ_{12} [MPa]	μ_{13} [MPa]	μ_{23} [MPa]	$\sigma_{\mathrm{Y}}^{\mathrm{ref}}$ [MPa]
2061	1728	1620	649	617	577	32
ν_{21} [-]	ν_{31} [-]	ν_{32} [-]	$\dot{\varepsilon}_{\mathrm{o}}[s^{-1}]$	C [-]	H^{ref}	σ_s [MPa]
0.39	0.39	0.28	1.0 e-4	0.06	0.05	$\sigma_Y(\theta)/1.15$

Supplementary Table 4: Orthotropic electrical and mechano-electrical parameters considered at the macroscopic scale and obtained from the optimisation procedure.

Electrical parameters			Mechano-electrical parameters			
$\hat{\Sigma}_{11}^{\mathrm{ref}}$ $[\frac{S}{m}]$	$\hat{\Sigma}_{22}^{\mathrm{ref}}$ $[\frac{S}{m}]$	$\hat{\Sigma}_{33}^{\mathrm{ref}}$ $[\frac{S}{m}]$	C_F^{11} [-]	C_{FdA} [-]	C_F^{22} [-]	C_F^{33} [-]
10.28	5.95	4.92	60	70	62	52

Supplementary Table 5: Thermal, thermo-mechanical and thermo-electrical parameters at the macroscopic scale, obtained from the optimisation procedure.

Thermal parameters			Thermo-mechanical parameters			Thermo-electrical parameter
k $[\frac{W}{mK}]$	ϱ $[\frac{J}{m^3K}]$	h $[\frac{W}{m^2K}]$	C_{Y} $[\frac{MPa}{^{\circ}C^2}]$	C_{E} $[\frac{MPa}{^{\circ}C^2}]$	C_{H} [-]	α_T $[^{\circ}C^{-1}]$
1.5	2.0 e6	10.8	0.025	1.35	-0.31	0.015

Additional mechano-electrical tests

This subsection presents additional experimental results of mechano-electrical tests. Firstly, the results for compressive mechano-electrical tests for the three principal directions configurations (longitudinal, transverse and vertical) are represented in Supplementary Fig. 7, with the aim of obtaining the maximum conductivity achieved in PLA/CB samples during compression. Secondly, the results for tensile mechano-electrical tests including the vertical printing orientation are presented in Supplementary Fig. 8.

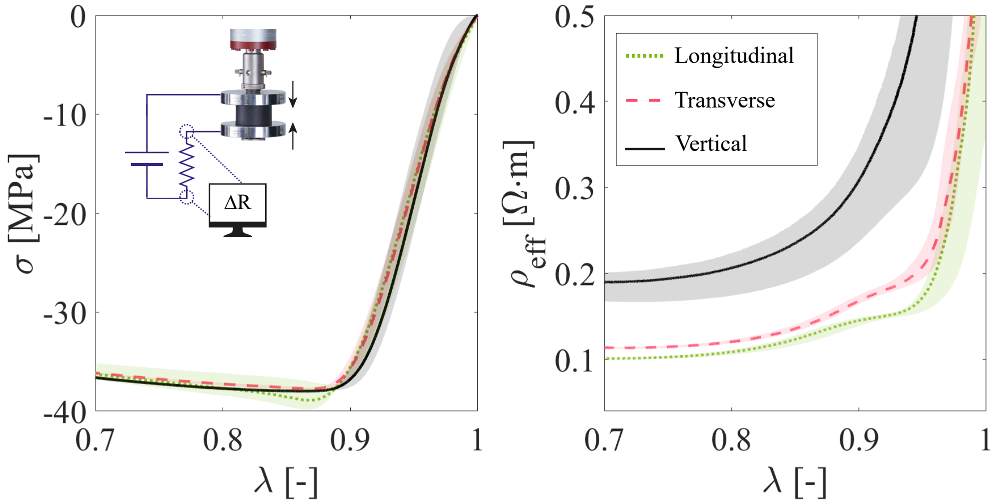

Supplementary Figure 7. Mechano-electrical compressive tests of PLA/CB samples. Uniaxial compressive tests were carried out while measuring the electrical current flowing through the samples. The solid/dashed lines represent the average values of the response, whereas the shaded areas represent the experimental deviation. Three samples were considered for each experimental condition. The directions considered for the tests are: longitudinal ($\mathbf{l}_1$), transversal ($\mathbf{l}_2$) and vertical ($\mathbf{l}_3$). A) The Cauchy stress is represented versus stretch. B) The effective resistivity is represented versus the mechanical stretch.

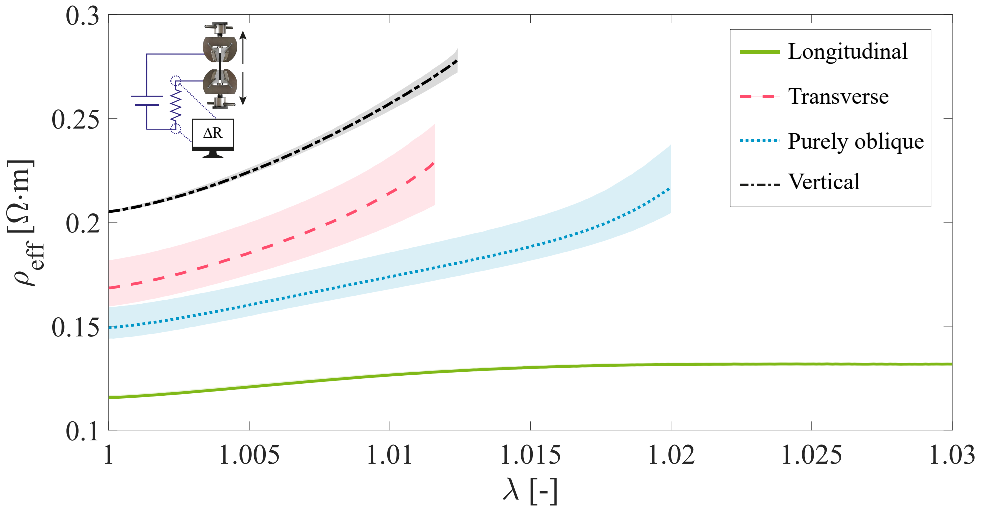

Supplementary Figure 8. Experimental results of mechano-electrical tensile tests performed to longitudinal, transverse, purely oblique and vertical samples. Uniaxial tensile tests were carried out while measuring the electrical current flowing through the samples. Three samples of each printing direction were employed during the testing. The solid/dashed lines represent the average of each orientation response, while the shaded area represents the experimental data distribution. The effective resistivity is represented versus the mechanical stretch.

Thermo-electro-mechanical experimental setup

This subsection provides a detailed overview of the experimental setup used for conducting thermo-electro-mechanical experiments, as depicted in Supplementary Fig. 9, along with the results obtained from fully coupled tests. The setup was designed to collect three types of data: (i) mechanical data (i.e. displacement and force), measured by using a universal testing machine (INSTRON 34TM-5, MA, USA); (ii) electrical data (i.e. voltage drop in a known resistor), measured by using a data acquisition system (DAQ DI-2008, OH, USA); and (iii) thermal data (i.e. surface temperature), measured by using an infrared camera (Fluke TiS75+, WA, USA). To ensure precise data collection, we synchronised the recording starting point of each machine by using a signal trigger which was controlled by an Arduino board (Arduino UNO, NY, USA). To measure the electrical data accurately, the DAQ system was used to measure the voltage values. For this purpose, a 1 Ω resistor was connected in series, and the voltage drop was measured through this known resistor. By using a resistor with a much lower resistance value in comparison to the sample's one, we ensured a correct heating without an interference from the additional resistor.

The proposed fully coupled experiment consisted of two steps. In the first step, an electro-thermal test was conducted, in which a constant electric field of 187.5 V/m was applied using a DC power source, resulting in an increase of the temperature of the filament. Both the infrared camera and the DAQ system were used to collect data during this step. In the second step, five minutes after the beginning of the experiment, a displacement ramp was applied to the filament while maintaining the applied electric field. All three machines were used to collect data during this step.

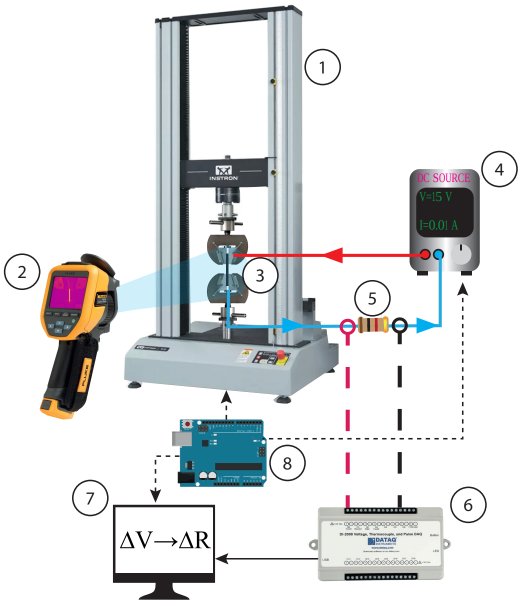

Supplementary Figure 9. Complete experimental set-up diagram for thermo-electro-mechanical tests. 1) Universal testing machine; 2) Infrared camera; 3) PLA/CB filament sample; 4) DC power source; 5) Known resistor; 6) DAQ system; 7) Computer, recording voltage data from DAQ system; 8) Signal trigger to control the electrical and mechanical recording

Heatable cartridge, thermal images and model data post-processing

This subsection provides an overview of the numerical and experimental post-processing of the temperature data taken from the heatable cartridge. As defined in the main manuscript, section "Smart selector of printing parameters to design multi-functional structural components", an optimal set of printing directions was sought to minimize the heating heterogeneity of the component. To this end, the standard deviation of the nodal temperatures of the cartridge was defined as the objective function to minimize. In this case, every node in the mesh was considered to calculate the temperature standard deviation. Nonetheless, to correctly compare the numerical results with the thermal IR images, a different set of nodes was selected. As shown in Supplementary Fig. 10.B, the IR images are taken from one side of the heating cartridge. Therefore, we only used the side surface nodes to compute the average and the

standard deviation of the temperatures during the heating process.

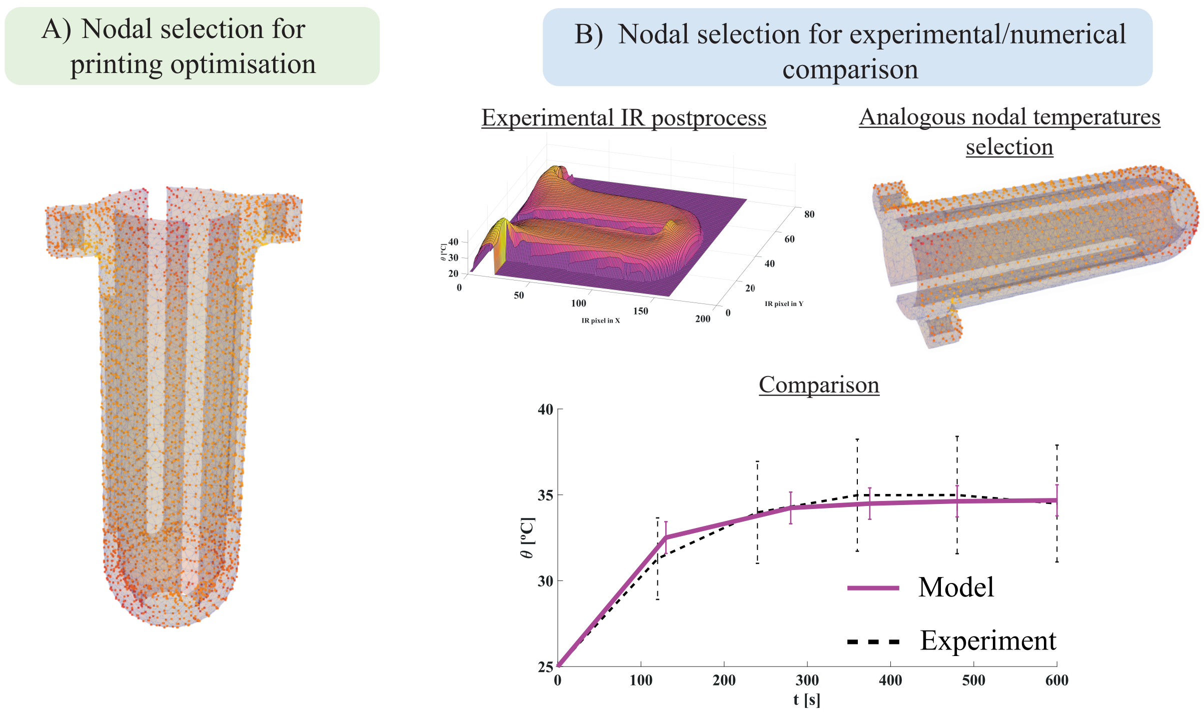

Supplementary Figure 10. A) Nodal selection of the whole mesh to optimise the heatable cartridge printing. B) Nodal selection of the side visible nodes, to correctly compare the IR image data with the numerical solution.

References

1. Barba, D., Arias, A. & Garcia-Gonzalez, D. Temperature and strain rate dependences on hardening and softening behaviours in semi-crystalline polymers: Application to PEEK. *Int. J. Solids Struct.* **182-183,** 205–217 (2020).
2. Crespo-Miguel, J., Lucarini, S., Arias, A. & Garcia-Gonzalez, D. Thermo-electro-mechanical microstructural interdependences in conductive thermoplastics. *npj Comput Mater* **9,** 134 (2023).
3. Itskov, M. A generalized orthotropic hyperelastic material model with application to incompressible shells. *Int. J. Numer. Methods Eng.* **50,** 1777–1799 (2001).
4. Itskov, M. & Aksel, N. A class of orthotropic and transversely isotropic hyperelastic constitutive models based on a polyconvex strain energy function. *Int. J. Solids Struct.* **41,** 3833–3848. ISSN: 0020-7683 (2004).
5. English, S. A. & A.Brown, A. *A 3D Orthotropic Elastic Continuum Damage Material Model* tech. rep. (Sandia National Laboratories, 2013).
6. Bing, Y., Garcia-Gonzalez, D., Voets, N. & Jérusalem, A. Medical imaging based in silico head model for ischaemic stroke simulation. *"J. Biomed. Mater. Res.".* **101,** 103442. ISSN: 1751-6161 (2020).
7. Hill, R. Elastic properties of reinforced solids: Some theoretical principles. *J. Mech. Phys. Solids* **11,** 357–372 (1963).
8. Geers, M. G. D., Kouznetsova, V. G., Matouš, K. & Yvonnet, J. in *Encyclopedia of Computational Mechanics Second Edition* 1–34 (John Wiley Sons, Ltd). ISBN: 9781119176817.
